# Anatomical description of the extratemporal facial nerve under high-definition system: a microsurgical study in rats

**DOI:** 10.1590/acb370803

**Published:** 2022-10-28

**Authors:** Marcela Maria Rabelo Pinto, Deivid Ramos dos Santos, Lívia Guerreiro de Barros Bentes, Rafael Silva Lemos, Nyara Rodrigues Conde de Almeida, Manuela Rodrigues Neiva Fernandes, Joyce Pantoja Braga, Danusa Neves Somensi, Rui Sergio Monteiro de Barros

**Affiliations:** 1 MD, Fellow Master Degree. Universidade do Estado do Pará – Postgraduate Program in Surgery and Experimental Research Medicine – Department of Experimental Surgery – Belem (PA), Brazil.; 2 Graduate student. Universidade do Estado do Pará – School of Medicine – Department of Experimental Surgery – Belém (PA), Brazil.; 3 Graduate student. Universidade Federal do Pará – School of Medicine – Department of Experimental Surgery - Belém (PA), Brazil.; 4 MD. Universidade Federal do Pará – School of Medicine – Department of Neurology – Belém (PA), Brazil.; 5 PhD, Associate Professor. Universidade Federal do Pará – School of Medicine – Department of Experimental Surgery – Belém (PA), Brazil.

**Keywords:** Facial Nerve, Facial Paralysis, Microsurgery, Microdissection, Anatomy, Rats

## Abstract

**Purpose:**

To describe the microsurgical anatomical aspects of the extratemporal facial nerve of Wistar rats under a high-definition video system.

**Methods:**

Ten male Wistar rats (12–15 weeks old), without veterinary diseases, weighing 220–280 g, were used in this study. All animals in this study were submitted to the same protocol and by the same surgeon. A 10-mm incision was made below the bony prominence of the right or left ear, and extended towards the angle of the mandible. The dissection was performed and the main branches of the facial nerve were dissected.

**Results:**

The main trunk of the facial nerve has a length of 0.88 ± 0.10 mm and a length of 3.81 ± 1.03 mm, measured from its emergence from the stylomastoid foramen to its bifurcation. Seven branches originating from the facial nerve were identified: posterior auricular, posterior cervical, cervical, mandibular, buccal, temporal, and zygomatic.

**Conclusions:**

The anatomy of the facial nerve is comparable to that of humans, with some variations. The most observed anatomical division was the distribution in posterior auricular, posterior cervical, cervical, mandibular, buccal, temporal, and zygomatic branches. There is no statistical difference between the thickness and distance of the structures compared to the contralateral side.

## Introduction

Facial paralysis affects over 40,000 individuals per year in the United States and can lead to permanent functional disfigurement, even with attempts at surgical intervention^1^^,^ 2. It usually leads to progressive deterioration of motor and sensory function, and consequently a considerable impact on the quality of life^3^^,^ 4. Moreover, it requires monitoring by medical specialists and physical therapy for functional recovery, imaging tests, and surgical treatment in up to 10% of the cases^5^^–^ 7. This condition has homogeneous distribution in all age groups worldwide, and despite its clinical importance, the cause is unknown in more than 60% of patients often reversible spontaneously; however, a significant proportion require a clinical or surgical approach^7^.

Some studies show that 20% of patients develop a kind of sequelae, such as complete paralysis of the facial muscles. In addition to their functional problems, patients suffering from facial paralysis are aware of their disfigurement and show deep psychological distress^1^^–^ 5.

Despite the development of new techniques, there is still no method to replace completely and satisfactorily the function of mimetic muscles^6^^,^ 7. Thus, every effort should be made to preserve or restore the neuromuscular units of the nerve-mimetic facial muscles promptly 8 ^–^ 12.

To support the development of new techniques aimed at improving the functional recovery of surgically treated injured facial nerves, experimental studies have been conducted seeking to understand the mechanisms involved in neural recovery^11^^–^ 16.

In the field of experimentation, the rat stands out as an important model used in studies involving nerves due to its easy acquisition and handling, not requiring large physical spaces, and few complications secondary to the surgical procedure are reported. It is also noteworthy that this model has great anatomical similarities with humans, allowing various comparisons^17^^,^ 18, such as the techniques of end-to-end neurorrhaphy in cases of neural gaps or performing autografts^19^.

Despite the great importance of the rat as a model for animal experimentation, facial nerve studies still lack microsurgical anatomical details. This gap needs to be filled given the wide use of these animals in research involving surgical repair in facial nerve models of injuries^12^^–^ 14.

This shows a still poorly explored field and the scarcity of information on this subject, which can support experimental research and contribute to the understanding of research involving humans. Knowing that this study will have a strong impact on the field of experimental research, this paper aims to describe the microsurgical anatomical aspects of the extratemporal facial nerve of Wistar rats.

## Methods

The study followed the rules established in the Brazilian national legislation on animal care (Law No. 11,794/2008), which is based on the US National Institutes of Health (NIH) guidelines, and complied with the code of ethics for animal experimentation of the International Organization of Medical Sciences Council and Animal Research: Reporting of In Vivo Experiments (ARRIVE) guidelines. The project was previously approved by the University Animal Care and Use Committee from Pará State University (protocol No. 18/2022).

### Experimental procedure

Ten male Wistar rats (12–15 weeks old), without veterinary diseases, weighing 220–280 g, were used in this study. All animals in this study were submitted to the same protocol and by the same surgeon. The animals were anesthetized with ketamine 60 mg/kg, xylazine 8 mg/kg, intraperitoneally^17^.

To confirm anesthesia, the anterior and posterior limb withdrawal reflex and the eyelid and tail reflexes were observed using forceps. Surgical anesthetic depth was defined when the animals lost at least three of the four reflexes (anterior paw withdrawal, posterior paw withdrawal, caudal, and vibrissae)^11^.

Once anesthesia was confirmed, the facial region was epilated. Antisepsis was performed with chlorhexidine. The animals were placed in a horizontal lateral decubitus position above a plastic stand. Euthanasia was then performed with an anesthetic overdose immediately before starting the surgical procedure to avoid drying of the neural tissue after the death of the animal.

**Figure 1:**
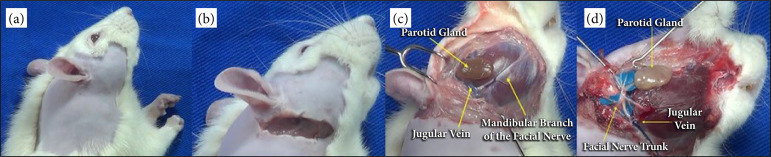
Dissection of the facial nerve of rats viewed laterally from the right; **(a)** Epilation; **(b)** Incision; **(c)** Identification of the parotid gland and jugular vein; **(d)** Dissection of the branches of the facial nerve and ligation of the jugular vein.

Initially, a small 10-mm incision was made just below the bony prominence of the right or left ear, depending on the side approached, and extended towards the angle of the mandible, carefully. Gentle dissection was performed and the main branches of the facial nerve were identified under direct visualization, the parotid gland can also be visualized, dissected, and extracted from the surgical field to facilitate visualization of the branches of the facial nerve (Fig. 1).

The distal branches of the facial nerve were identified just below the parotid. These are followed proximally to the main trunk of the facial nerve. Once identified, the main trunk and the upper and lower bifurcation of the facial nerve are carefully dissected.

The morphometric analysis was performed by obtaining the length (millimeters) measured from the lateral vertebrae body border identified during each dissection.

The procedures were performed under a video system composed by a high-definition Sony camcorder HDR-CX 150 set to 52× magnification, connected with a 4K 55-inch curved television, positioned in front of the surgeon, by a digital HDMI cable. Two white led light sources were used near the camera to provide adequate illumination of the operative field^11^^,^ 14.

During the video documentation, a millimeter paper was used as a reference for the measurements of the morphometric analysis. To measure the structures, the Image J software was used.

BioEstat 5.4 software was used. Student’s t test was used to compare the study variables. Statistical significance was assumed at p < 0.05. All data were expressed as means ± standard deviation.

## Results

### Origin and branches of the facial nerve

**Figure 2:**
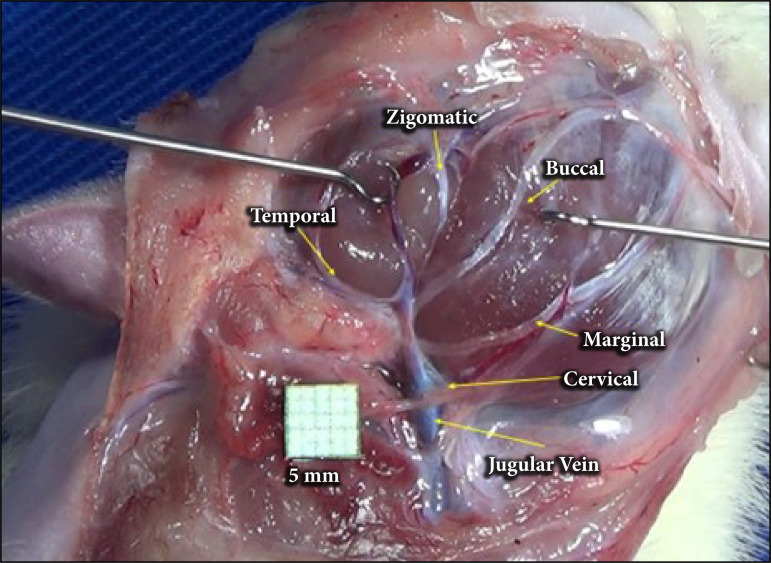
Anatomical arrangement of the facial nerve branches afterremoval of the parotid gland for better visualization of the nerves.

**Figure 3:**
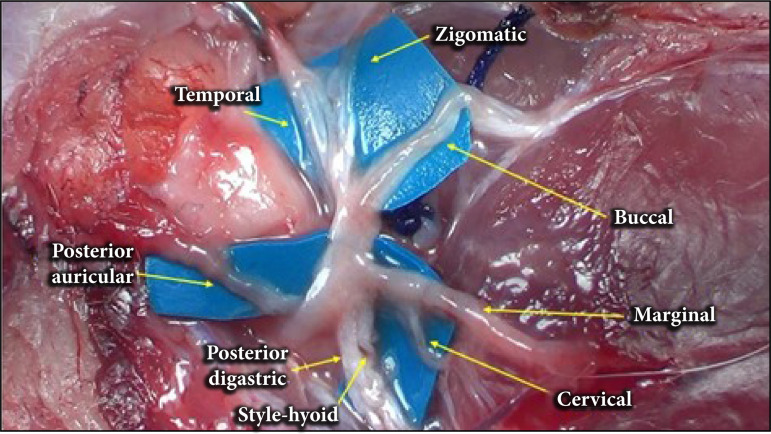
Anatomical arrangement of the facial nervebranches after removal of the parotid gland forbetter visualization of the nerves.

The extratemporal facial nerve originates at the stylomastoid foramen, located lateral to the skull, posterosuperior to the external acoustic meatus (Fig. 2). The main trunk of the facial nerve has a length of 0.88 ± 0.10 mm and a length of 3.81 ± 1.03 mm, measured from its emergence from the stylomastoid foramen to its bifurcation (Table 1). Seven peripheral branches originating from it were identified: posterior auricular, posterior cervical, cervical, mandibular, buccal, temporal, and zygomatic (Fig. 3).

**Table 1:** Measuring the distance of agreed-upon points from the facial nervetrunk after emerging from the external acoustic meatus.

Facial Nerve	Average (mm) ± standard deviation		
	Right	Left	Average
Posterior auricular – Length	6.65 ± 0.94	6.57 ± 0.83	6.61 ± 0.88
Posterior auricular – Diameter	0.36 ± 0.08	0.32 ± 0.10	0.34 ± 0.90
Facial nerve – Length	3.88 ± 1.02	3.75 ± 1.05	3.81 ± 1.03
Facial nerve – Diameter	0.91 ± 0.10	0.85 ± 0.10	0.88 ± 0.10
Posterior cervical nerve – Diameter	0.22 ± 0.08	0.26 ± 0.06	0.24 ± 0.07
Mandibular – Length	18.16 ± 0.72	18.23 ± 1.15	18.19 ± 0.93
Mandibular – Diameter	0.56 ± 0.10	0.49 ± 0.21	0.52 ± 0.15
Buccal – Length	24.32 ± 1.58	23.20 ± 1.06	23.76 ± 1.32
Buccal – Diameter	0.40 ± 0.06	0.47 ± 0.04	43.50 ± 0.05
Zygomatic – Length	11.50 ± 0.80	10.72 ± 0.92	11.11 ± 0.86
Zygomatic – Diameter	0.45 ± 0.04	0.49 ± 0.06	0.47 ± 0.05
Temporal – Diameter	0.19 ± 0.04	0.20 ± 0.04	0.19 ± 0.04

Mandibular: trunk length up to the marginal division; Buccal: trunk length to the masseter division; Zygomatic: trunk length up to the bifurcation; Temporal: length not measured due to anatomical variations in bifurcations.

### Branches

The posterior auricular branch has its origin immediately after the emergence of the facial nerve through the stylomastoid foramen (Fig. 3). The posterior cervical branch follows laterally to the external jugular vein. The cervical branch is posteroinferior, located deep to the jugular vein. The mandibular branch follows its path through the central of masseter muscle. The buccal branch originates between the masseter muscle and the temporal branch. This, in turn, runs superiorly to the eye. The zygomatic branch has two branches that anastomose to different nerves.

### Distal branches

**Figure 4:**
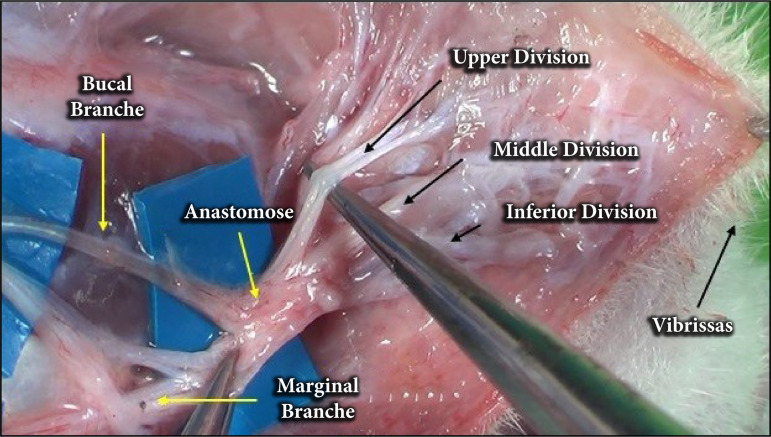
Anatomical division of the buccal nerve anastomosiswith the marginal nerve forming the superior, middle,and inferior branches (distal pass).

The buccal and marginal branches of the facial nerve distally anastomose to form the distal feet, which are divided into an upper, middle, and lower branch, innervating the vibrissae (Fig. 4).

## Discussion

### Experimental model and videomagnification system

Recent surgical advances have been described in the treatment of facial paralysis in humans, but the functional results are still unsatisfactory^12^^–^ 15. This leads to hypotheses being tested in experimental studies to find better solutions or improve neural repair techniques, especially about the best approach and surgical treatment time.

One of the most used models is the rat (Wistar), which has several peculiarities similar to humans, making it one of the main species used in experimental studies on peripheral nerves, mainly due to its anatomical and functional similarity^18^^,^ 19.

Although this experimental model is used in several studies on facial nerves, few of them consider microsurgical anatomy and possible anatomical variations, which may lead to significant biases in interpretation due to the different neural arrangements present in the model in question. Specific studies on the anatomy of the facial nerve of rats are old and lacked the technology to microdissect the terminal branches of the nerve and understand their anatomical distribution^20^^,^ 21. For this, the extended video microsurgery system was used, which allows greater comfort for the surgeon and better access to the surgical field, making dissection less time-consuming^19^.

It is not uncommon to find studies that show the difference in the anatomical and functional distribution of the peripheral nerves of rats compared to human anatomy, in the sense that a nerve has a specific function in humans and does not perform the same function in the experiments^19^^,^ 25. It is important to emphasize that the pathway of the peripheral nerves in rats is not the same in humans.

It is important to emphasize that the access route to the facial nerve is superficial, but requires the skills of an experienced surgeon, due to a large amount of fat between the nerves. When using videomagnification, the third dimension is lost, increasing the degree of difficulty in manipulating small structures, which does not occur in conventional microscopy.

### Facial nerve

Some authors describe the anatomy of rats used in experimental work without, however, demonstrating the microsurgical details of the terminal branches of the peripheral nerves^11^^–^ 16. Anatomically, the extratemporal facial nerve of Wistar rats emerges from the stylomastoid foramen, located laterally in the skull. The first branch, posterior auricular, with the function of innervating the auricular musculature, emerges just after the foramen; this makes it difficult to dissect it without injuring part of it, due to its intrinsic relationship with the adjacent tissue.

The facial nerve trunk measures approximately 3.81 ± 1.03 and 0.80 ± 0.10 mm in diameter, being thinner than described, which length at the time was 6 mm. The nerve is easily seen, having a path between the trapezius muscle and the auditory canal. The mandibular branch has a bifurcation at its distal end ending in branches that innervate the muscles of the lower lip^23^^–^ 25.

This makes it difficult to dissect it without damaging part of it, due to its intrinsic relationship with the adjacent tissue. Some authors report the need to remove part of the temporal bone to facilitate surgical access^20^^–^ 22, but in this study the cranial bones were preserved. It is noteworthy that rats do not have an internal jugular vein; thus, all cranial drainage occurs through tributary veins of the external jugular vein, which has a clear path laterally to almost all branches that make up the facial nerve^26^^,^ 27.

The temporal and zygomatic branches emerge directly from the trunk of the facial nerve in 80% of the sample studied. In one animal, the branches emerged directly from the buccal nerve. This anatomical variation is in line with what has already been described in the literature, justifying why these two branches are not used in experimental studies. Moreover, they have a complex pattern of distribution with variation in the pattern of ramifications, making it difficult to perform surgical procedures, especially in the zygomatic branch of the facial nerve^20^^,^ 21.

In this study, the temporal and posterior cervical branches of the facial nerve had the smallest diameter and their lengths were not measured because of the anatomical variation of their subdivisions. Some studies state that the posterior cervical nerve constitutes the shortest length^20^^,^ 21.

It is possible to observe that the cervical nerve has a posteroinferior path in comparison to the other branches. The buccal nerve, follows between the masseter and temporalis muscles, passing inferiorly to the eyes in the nasal direction. A study reports that the final divisions of the mandibular branch in Wistar rats innervate both muscle fibers of the lower and upper lip^20^. They also mention that the infratemporal portion of the facial nerve in rats is purely motor, resembling other peripheral nerves^23^^,^ 28 ^,^ 29. The nerve stimulation was not performed to determine the sensory and motor function, configuring one of the biases of this study.

The pattern of facial nerve division in rats is similar to that found in rodents. It is noteworthy that the presence of several anatomical variations not yet studied may not accurately reflect the desired human clinical situation in experimental studies. Even so, in addition to the anatomical similarity with several mammals, the use of the facial nerve of rats offers clear ease of dissection, since the nerves rest on the facial muscle fascia in the subcutaneous cellular tissue, facilitating dissection at specific points. The branches of the rat facial nerve pass below the parotid gland and are not across as found in other rodents.

The mandibular ramus crosses the masseter muscle inferiorly and has a length of 18.19 ± 0.93 mm and a diameter of 0.52 ± 0.15 mm, as found in other studies^8^^,^ 9. These dimensions allow the use of this segment in experimental research on end-to-end repair or autologous nerve grafting. The authors found no statistically significant differences between sides (p = 0.248; p = 0.533) or between proximal and distal segments (p = 0.248; p = 0.533), proximal and distal segments (p = 0.859; p = 0.182).

## Conclusion

The anatomy of the facial nerve is comparable to that of humans, with some variations. The most observed anatomical division was the distribution in posterior auricular, posterior cervical, cervical, mandibular, buccal, temporal, and zygomatic branches. There is no statistical difference between the thickness and distance of the structures compared to the contralateral side.
